# Social withdrawal subtypes and psychological well-being in Chinese emerging and early adults: unsociability as a protective factor and age-differentiated effects in a new media social environment

**DOI:** 10.3389/fpsyt.2025.1671609

**Published:** 2025-10-20

**Authors:** Tomoko Kishimoto, Tianyu Wang, Qiyu Bai

**Affiliations:** ^1^ Faculty of Psychology, Beijing Normal University, Beijing, China; ^2^ Department of Social Psychology, Nankai University, Tianjin, China; ^3^ School of New Media, Peking University, Beijing, China

**Keywords:** social withdrawal, unsociability, emerging and early adulthood, psychological well-being, age moderation

## Abstract

**Introduction:**

In the digital age, social withdrawal as a stable personality trait has become increasingly complex, as individuals may engage in face-to-face withdrawal while maintaining digital social connections through new media platforms. Well-established withdrawal subtypes have been studied in Western cultures, but their implications in Chinese digital social contexts remain underexplored.

**Methods:**

This cross-sectional study examined the associations between different social withdrawal subtypes and psychological well-being indices among Chinese emerging and early adults, with consideration of the contemporary digital social landscape. Participants (n = 1365, M_age = 27.79) completed an online survey including the Social Preference Scale for Adult-Chinese Revised (SPSA-CR), measures of psychological well-being, and relationship satisfaction.

**Results:**

Results showed that each social withdrawal subtype was differentially associated with psychological well-being indices: shyness was significantly associated with internalizing problems, externalizing problems, and relationship satisfaction; avoidance was significantly associated with internalizing problems and relationship satisfaction; unsociability was significantly associated with aggression and relationship satisfaction. Age moderated the associations between shyness and psychological well-being indices, though effect sizes were small (β = -0.072 to 0.133).

**Discussion:**

These cross-sectional findings suggest differential associations between withdrawal subtypes and well-being in Chinese cultural contexts.

## 1 Introduction

### 1.1 Subtypes of social withdrawal: a multidimensional framework

Social withdrawal refers to a consistent tendency to disengage from social interaction and seek solitude. While withdrawal has traditionally been treated as a unidimensional risk factor for maladjustment, contemporary models have identified at least three functionally distinct subtypes: shyness (withdrawal motivated by social anxiety), avoidance (active withdrawal motivated by discomfort or dislike of others), and unsociability (a preference for solitude without social fear) (1, 2). These subtypes differ not only in motivation and behavioral expression, but also in their psychological correlates and developmental implications (3, 4).

Shyness and avoidance have consistently been linked to negative outcomes such as internalizing symptoms, peer rejection, and lower well-being (2, 5). By contrast, unsociability has demonstrated more complex associations: while traditionally viewed as a milder risk factor in Western literature, it may be developmentally neutral or even beneficial under certain cultural or contextual conditions. Importantly, most of this literature has focused on children and adolescents, with fewer studies extending into adult populations, particularly in non-Western cultural contexts.

With the rise of new media and digital platforms, social interaction patterns among emerging adults have undergone profound changes. Individuals can maintain social presence without face-to-face interaction, and solitary behaviors may increasingly occur within digitally connected environments. This evolution blurs the lines between physical social withdrawal and online social engagement. Therefore, examining subtypes of social withdrawal in the digital age requires considering the role of new media in shaping both behavior and its psychological consequences (6, 7).

### 1.2 Cultural context: rethinking unsociability in China

Although the subtypes of social withdrawal have been well-documented in Western contexts, their manifestations and psychological implications may vary considerably across cultures. This is especially true for unsociability, which is marked by a low social approach motivation rather than social anxiety or aversion. In individualistic societies, where autonomy and self-expression are highly valued, individuals who display a strong preference for solitude—especially the unsociable—are often perceived as deviant or at risk for maladjustment. In collectivistic cultures such as China, however, the picture is more nuanced. On the one hand, overt withdrawal from group interaction can be viewed as counter-normative, threatening interpersonal harmony and group cohesion. On the other hand, solitude that reflects emotional restraint, self-discipline, or intellectual maturity—values deeply rooted in Confucian heritage—may be interpreted more leniently, especially when it does not lead to interpersonal conflict or disrupt collective functioning. This nuanced view is supported by a cultural tradition that positively frames chosen solitude. Beyond the Confucian emphasis on self-cultivation (e.g., the concept of Shendu, or “being careful when alone”), Taoist philosophy also highlights the value of controlling desires and engaging in self-reflection during solitary time (8). Furthermore, cultural adages such as “自古圣贤皆寂寞” (“All sages have been lonely since ancient times”) suggest that a preference for solitude can be associated with intellectual rewards and respect (9). As such, from a young age, individuals in Chinese contexts may be socialized to view certain forms of solitude as opportunities for self-improvement rather than merely as social disengagement. Thus, the social meaning of solitude is not uniform across contexts, and the motivational attributions behind unsociability become critical in determining whether it is deemed adaptive or problematic.

Recent cross-cultural and regional studies provide empirical support for these cultural dynamics. A study of Greek emerging adults found that unsociability was negatively associated with existential anxiety, suggesting it may function as a protective factor in some adult Western samples (10). In contrast, research on Chinese preschool migrant children revealed that unsociability predicted peer exclusion and internalizing symptoms only when resilience was low, pointing to the role of individual moderators in determining outcomes (11). A recent study among Chinese college students also found that depression mediated the association between social withdrawal subtypes and sleep problems, but this pathway was not significant for unsociability (12), further highlighting its motivational distinctiveness and potential adaptive role.

Taken together, these findings suggest that the implications of unsociability are culturally fluid and developmentally contingent. Although the present study does not include a direct cross-cultural comparison, it contributes to this growing body of work by examining unsociability’s link to well-being among Chinese emerging and early adults—a population rarely studied in this context. By embedding our inquiry within a specific cultural framework, we aim to clarify how solitude-seeking behavior may serve adaptive, neutral, or maladaptive functions depending on societal expectations and developmental timing.

### 1.3 Developmental perspectives: theoretical basis for age-moderated effects

Building on the notion that the implications of unsociability are culturally and developmentally contingent, it is crucial to consider how such behaviors are interpreted across different stages of adulthood. Emerging and early adulthood, roughly spanning ages 18 to 35, represents a period of intensified identity exploration, shifting social roles, and increasing demands for both autonomy and relational competence (13, 14). While the original concept of emerging adulthood focused primarily on individuals in their late teens to twenties, developmental scholars have increasingly acknowledged that many key features of this phase—such as role ambiguity, instability in work and relationships, and delayed transition to traditional adulthood markers—often extend into the early thirties, especially in East Asian societies (15).

In the Chinese context, this prolonged transition is culturally embedded in the idea of “三十而立” (“thirty as the age of establishment”), a Confucian ideal that reflects both societal expectations and internalized developmental milestones. Adulthood is not viewed as a discrete threshold but as a gradual consolidation of autonomy, stability, and responsibility. From this perspective, solitude-seeking behaviors like unsociability may be interpreted differently depending on age-related expectations. For example, a 22-year-old who prefers solitude might be perceived as developmentally behind, whereas a 32-year-old exhibiting the same behavior could be seen as reflective or mature—provided their behavior aligns with cultural norms around self-regulation and independence.

This interpretation aligns with the developmental timing hypothesis (3), which posits that the psychosocial implications of social withdrawal vary by life stage. While unsociability in childhood is often linked to social immaturity or peer exclusion, in adulthood—particularly within certain cultural frameworks—it may signal autonomy, self-sufficiency, or adaptive emotional regulation.

Recent research adds further nuance. Yuan et al. (12) found that depressive symptoms mediated the relationship between shyness or avoidance and sleep disturbances, but not between unsociability and sleep—indicating that unsociability may be less emotionally burdensome in adult samples. These findings support the possibility that the well-being outcomes of different withdrawal subtypes are age-sensitive, and that unsociability may become increasingly adaptive or benign with age, especially in cultures that value introspection, stability, and emotional control.

### 1.4 Research aims and hypotheses

Despite the growing recognition that social withdrawal is a multidimensional construct, most existing research has focused on children and adolescents, leaving important gaps in our understanding of how withdrawal subtypes function in adulthood—particularly in non-Western cultural contexts. Moreover, few studies have examined how age-related developmental transitions shape the psychological implications of these subtypes during emerging and early adulthood, a period marked by identity formation, shifting role demands, and culturally embedded expectations for independence.

To address these gaps, the present study investigates the associations between three core subtypes of social withdrawal—shyness, avoidance, and unsociability—and key indicators of psychological well-being among a sample of Chinese emerging and early adults (aged 18–35). By embedding this inquiry within a cultural-developmental framework, we aim to clarify when, and for whom, solitude-seeking behavior may be adaptive, neutral, or maladaptive in adulthood.

Guided by the developmental timing hypothesis (3), Eriksonian psychosocial theory, and culturally embedded age norms (e.g., “thirty as the age of establishment”), we propose the following hypotheses:

- H1: Shyness and avoidance will be negatively associated with psychological well-being, consistent with prior evidence linking these subtypes to anxiety, fear-based withdrawal, and emotional distress.
- H2: Unsociability will show a non-significant or positive association with psychological well-being, reflecting its motivational distinctiveness and potential cultural reappraisal in the Chinese context.
- H3: Age will moderate the relationship between unsociability and well-being, such that the association becomes increasingly positive (or less negative) in later stages of emerging and early adulthood.

By explicitly testing these hypotheses, this study aims to clarify the nuanced role of solitude in mental health and challenge overly pathologized views of social withdrawal, particularly in non-Western adult populations. Findings may also inform culturally and developmentally tailored psychological interventions that recognize the diversity of withdrawal motivations and meanings.

## 2 Methods

### 2.1 Participants and procedure

The sample size was 1365, aged 18 to 35 (M_age_ = 27.79, SD = 4.16, 51.6% female, see **Table 1**), meet the minimum sample size requirements for structural equation modeling (16). To collect data, we purchased a sample service from Wenjuanxing, a leading online survey platform in China. The platform distributed the survey link through its proprietary participant pool. Participants were invited via email and in-app notifications, and the survey was promoted to ensure a broad geographic and socioeconomic representation. Each participant received a reward of 18 yuan upon completion of the survey. The study received ethical approval from the Behavioral Research Ethics Board of local university.

**Table 1:** Descriptive characteristics of the study sample (N = 1365).

Variable	Category	n/M	%/SD
Age (years)		27.79	4.16
	18-24	286	21.00%
	25-29	577	42.20%
	30-35	502	36.80%
Gender	Female	705	51.60%
	Male	660	48.40%
Education Level	High school or below	111	8.10%
	Bachelor’s degree	1077	78.90%
	Master’s degree or above	177	13.00%
Annual Household Income (RMB)	< 80,000	142	10.40%
	80,000 - 150,000	417	30.50%
	150,000 - 300,000	566	41.50%
	> 300,000	240	17.60%
Marital Status	Single	503	36.80%
	Married/Cohabiting	856	63.10%

### 2.2 Measures

#### 2.2.1 Social withdrawal

We used the revised Chinese version of the Child Preference Scale adult revised (see Supplementary information) to measure social withdrawal. The adult version of the Child Preference Scale was revised by Nelson (2) which was compiled by Coplan et al. (17). This measure includes 14 items, and consists of three dimensions: avoidance (6-item, α = .81), shyness (5-item, α = .83), and unsociability (3-item, α = .68). Participants rated the items on a 5-point Likert scale (1 = “Not at all” to 5 = “A lot”). Internal consistency for this measure was good (α = .77).

#### 2.2.2 Relationship satisfaction

The Comprehensive Diagnostic Scale of Interpersonal Relationships compiled by Zheng (18) included 28 items and consisted of four dimensions: conversation, communication, dealing with people, and heterosexual communication. Subjects were asked to make a “yes (= 1) or no (= 0)” forced-choice response to each item. We use the reverse score of this scale to express the relationship satisfaction of the subjects. A higher score indicated higher satisfaction of the subjects. Internal consistency for this measure was good (α = .75).

#### 2.2.3 Anxiety

The Self-rating Anxiety Scale (SAS-CR) was compiled by Zung (19). We used the Chinese version revised by Tao and Gao (20) to measure anxiety symptoms. This measure includes 20-item. Five items are scored in reverse. Participants rated the items on a 4-point scale (1 = “Not at all” to 4 = “A lot”). Higher scores indicate higher levels of anxiety. Internal consistency for this measure was excellent (α = .93).

#### 2.2.4 Depression

The simplified version of the Center for Epidemiologic Studies Depression Scale (CES-D) was compiled by Kohout et al. (21). We used the Chinese version revised by He et al. (22) to measure depression symptoms. This measure includes 9-item. Participants rated the items on a 4-point scale, ranging from 0 = “Rarely or none of the time” (less than one day in the last week) to 3 = “Most or all the time” (5-7 days in the last week). Higher scores indicate higher levels of depression. Internal consistency for this measure was very good (α = .88).

#### 2.2.5 Aggression

The Buss & Perry Aggression Questionnaire (AQ-CV) was compiled by Buss and Perry (23). We used the Chinese version revised by Li et al. (24) to measure aggression. This measure includes 30-item, and consists of five subscales of physical aggression (7 items, α = .75), verbal aggression (5 items, α = .67), anger (6 items, α = .78), hostility (7 items, α = .70), and self-aggression (5 items, α = .68). Participants rated the items on a 5-point scale (1 = “Not at all” to 5 = “A lot”). Higher scores indicate higher levels of aggression. Internal consistency for this measure was very good (α = .89).

### 2.3 Statistical analysis

SPSS 26 and Mplus 8.3 were used in the statistical analyses. Firstly, Zero-order correlations were computed to examine bivariate associations between all variables. Secondly, latent variable modeling was used to analyze moderation models. We parceled the observation index items of the dependent variable within the dimension (25). We used Latent Moderated Structural Equations (LMS) to confirm the moderation role of age (26) and assessed the model fitting index according to criteria of Bentler (27). We first mean-centered the continuous data, then conducted moderation analyses using anxiety, depression, aggression, and relationship satisfaction as outcome variables, social withdrawal dimensions as independent variable, and age as moderator variable.

## 3 Results

### 3.1 Common method bias

To test for potential common method bias, we conducted Harman’s single factor test (28). The result shows, 21 factors had eigenvalues above 1, and 19.42% of the total variation was attributed to the first factor, below the threshold of 40%, indicating that common method bias was not a serious concern in this study.

#### 3.1.1 Descriptive statistics and correlations

Means, standard deviations, and zero-order correlations for all variables are presented in **Table 2**. The three social withdrawal subtypes showed significant positive correlations with anxiety, depression, and aggression, and negative correlations with interpersonal satisfaction.

**Table 2:** Means, standard deviations, and correlations for study variables (n = 1365).

Variable	1	2	3	4	5	6	7	8
1. age								
2. avoidance	-.163**							
3. shyness	-.262**	.423**						
4. unsociability	-.171**	.672**	.492**					
5. relationship satisfaction	.190**	-.422**	-.689**	-.446**				
6. anxiety	-.098**	.344**	.451**	.317**	-.584**			
7. depression	-.127**	.405**	.485**	.381**	-.586**	.641**		
8. aggression	-0.015	.218**	.383**	.291**	-.511**	.516**	.536**	
M	27.79	2.09	2.76	2.87	18.87	1.95	1.86	2.20
SD	4.16	0.70	0.95	0.88	5.46	0.45	0.60	0.60

** *p* < 0.01.

### 3.2 Effects of social withdrawal on well-being indices and the moderation role of age

**Figure 1:**
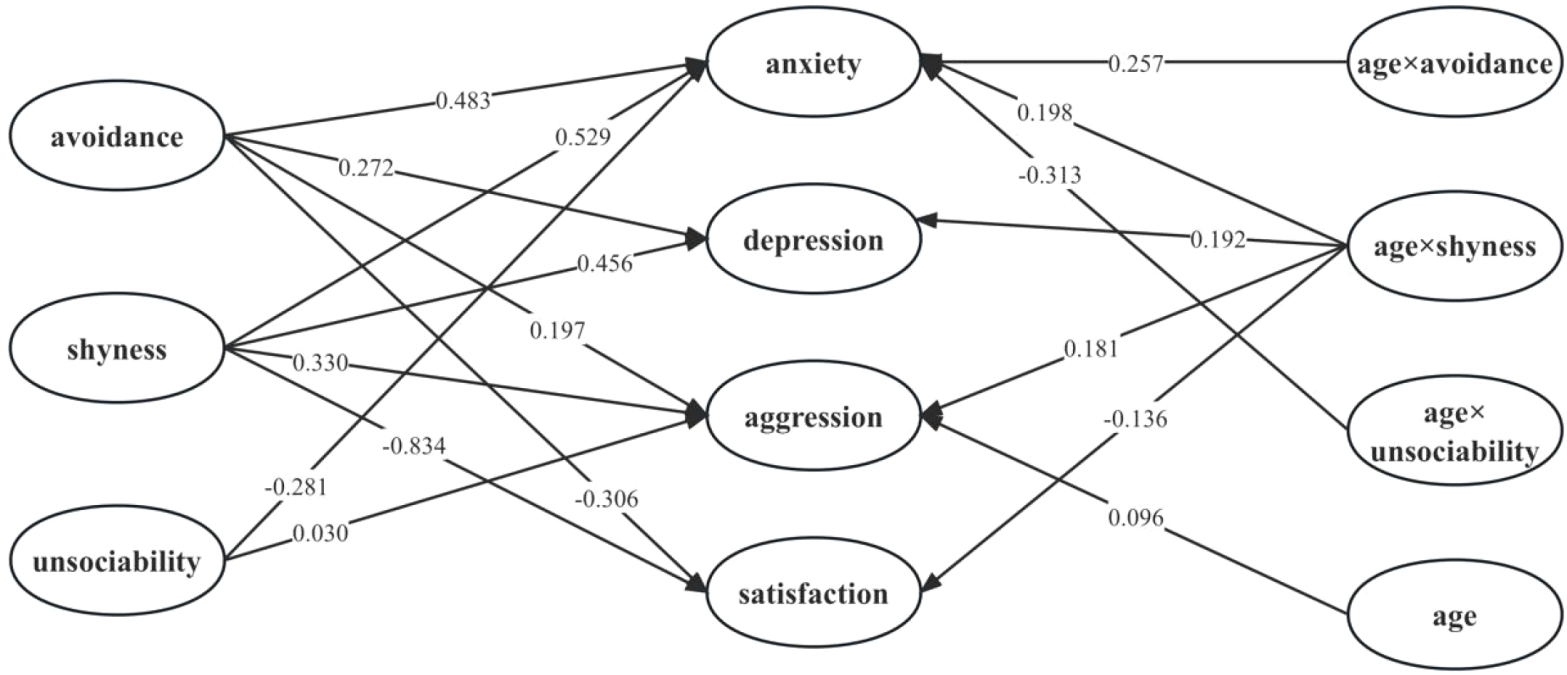
The moderation model of age on the relationship between social withdrawal dimensions and well-being indices.

The results show that the fit index of the model is good (CFI = 0.913, TLI = 0.904, RMSEA = 0.037, SRMR = 0.046), **Figure 1** shows the standardized coefficients of each path. The standardized path coefficients for all direct and moderation effects are presented in **Table 3**.

**Table 3:** Standardized path coefficients for the effects of social withdrawal subtypes on psychological well-being indices and the moderating role of Age (N = 1365).

Predictor	Anxiety*β*(SE)	Depression*β*(SE)	Aggression*β*(SE)	Relationship satisfaction*β*(SE)
Direct effects				
age	0.057(0.028)	0.040(0.026)	0.096***(0.026)	-0.012(0.022)
Shyness	0.529***(0.032)	0.456***(0.051)	0.383***(0.051)	-0.834***(0.042)
Avoidance	0.483***(0.029)	0.272***(0.126)	0.197***(0.041)	-0.306**(0.116)
Unsociability	-0.281*(0.142)	-0.205(0.150)	0.030***(0.007)	0.203(0.148)
Interaction effects				
age×Shyness	0.198***(0.024)	0.192***(0.022)	0.181***(0.023)	-0.136***(0.030)
age×Avoidance	0.257*(0.109)	0.106(0.102)	-0.029(0.095)	-0.138(0.095)
age×Unsociability	-0.313*(0.125)	-0.224(0.118)	-0.037(0.111)	0.189(0.112)

*p* <.05, **p* <.01, ***p* <.001.

Shyness was positively associated with anxiety, depression, and aggression positively (*β*_S_ = 0.330 to 0.529, *p*s < 0.001), predicted relationship satisfaction negatively (*β* = -0.853, *p* < 0.001), and age moderated the pathway from shyness to all indices of well-being (*β*_S_ = -0.136 to 0.198, *p*s < 0.001). Avoidance predicted anxiety, depression, and aggression positively (*β*_S_ = 0.197 to 0.483, *p*s < 0.001), predicted relationship satisfaction negatively (*β* = -0.306, *p* = 0.008), and age moderated the pathway from avoidance to anxiety (*β* = 0.257, *p* = 0.019). Unsociability predicted anxiety negatively (*β* = -0.281, *p* = 0.047), predicted aggression positively (*β* = 0.030, *p* < 0.001), and age moderated the pathway from unsociability to anxiety (*β* = -0.313, *p* = 0.012). The results showed that each withdrawal dimensions had unique link with the well-being indices and the influences of age were different in different dimension, which partly supported H2.

**Figure 2:**
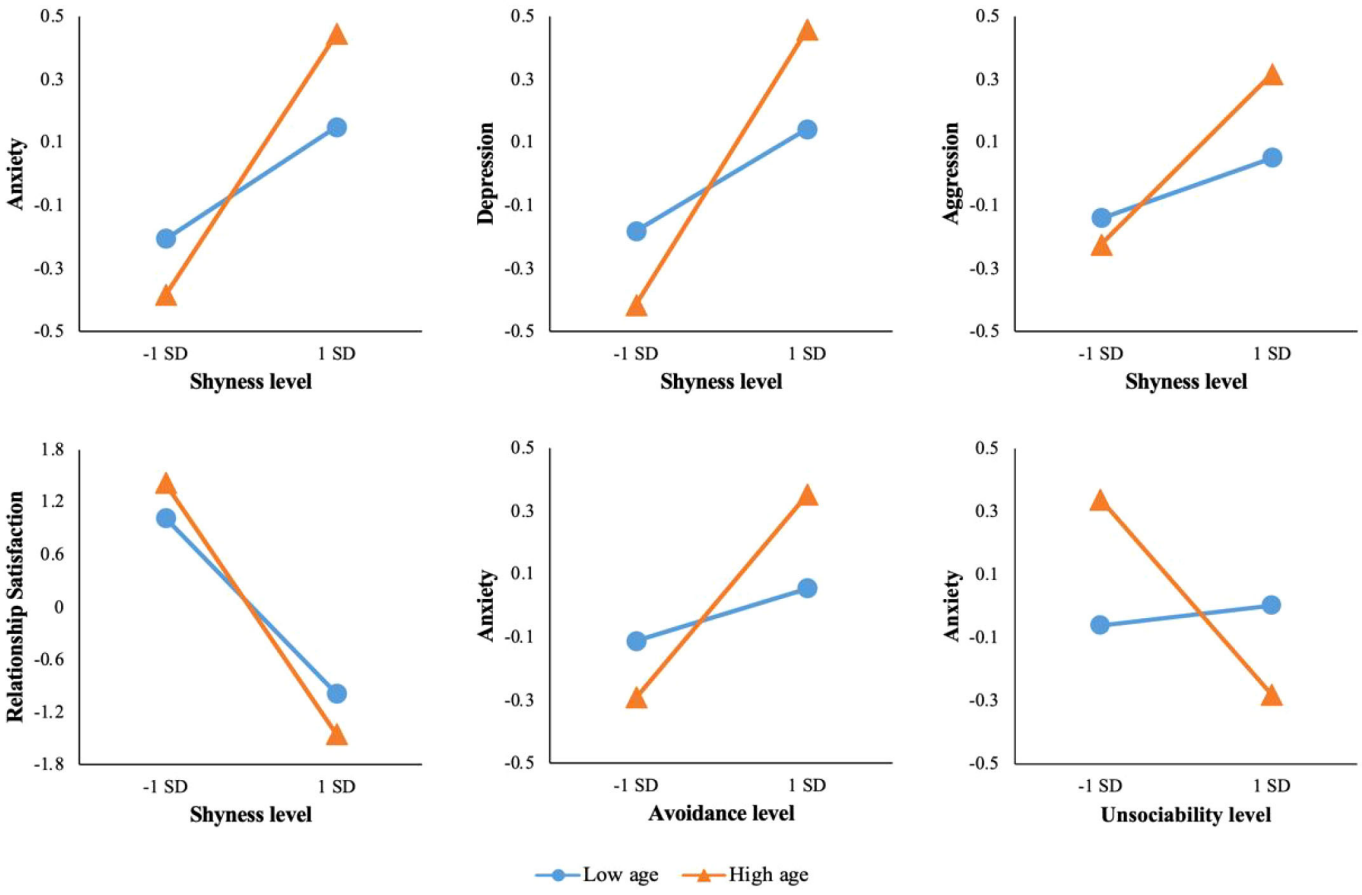
Simple slopes analyses of the interaction between age and shyness level.

Simple slope analyses were conducted to examine the associations between each well-being index and social withdrawal dimension at low (−1 SD below the mean, n = 238) and high (+1 SD above the mean, n = 268) levels of age. Participants within ±1 SD of the mean (n = 859, 62.9% of the sample) were included in the overall analysis but were not separately categorized for slope comparisons. (see **Table 4**, **Figure 2**).

**Table 4:** Simple slopes analysis: probing the interaction effects of age and social withdrawal subtypes.

Subtype	Outcome variable	At low age (-1 SD) B	At high age (+1 SD) B
Shyness	Anxiety	0.209***	0.494***
	Depression	0.182***	0.487***
	Aggression	0.080*	0.314***
	Relationship Satisfaction	-1.278***	-1.809***
Avoidance	Anxiety	0.264*	0.811***
Unsociability	Anxiety	0.047	-0.641*

The symbol "*" indicates marginal significance p < 0.05, and "***" indicates p < 0.001.

Shyness had a significant positive predictive effect on anxiety, depression and aggression at low levels of age (B_S-an_ = 0.209, p < 0.001; B_S-de_ = 0.182, p < 0.001; B_S-ag_ = 0.080, p = 0.005), the positive predictive effect of shyness on anxiety, depression and aggression was significantly enhanced at high levels of age (B_S-an_ = 0.494, p < 0.001, B_S-an_ changed from 0.209 to 0.494; B_S-de_ = 0.487, p < 0.001, B_S-de_ changed from 0.182 to 0.487; B_S-ag_ = 0.314, p < 0.001, B_S-ag_ changed from 0.080 to 0.314).

Shyness had a significant negative predictive effect on relationship satisfaction at low levels of age (B_S-RS_ = -1.278, p < 0.001), the negative predictive effect of shyness on relationship satisfaction was significantly enhanced at high levels of age (B_S-RS_ = -1.809, p < 0.001; B_S-RS_ changed from -1.278 to -1.809).

Avoidance had a significant positive predictive effect on anxiety at low levels of age (B_A-an_ = 0.264, p = 0.027), the positive predictive effect of avoidance on anxiety was significantly enhanced at high levels of age (B_A-an_ = 0.811, p < 0.001; B_A-an_ changed from 0.264 to 0.811).

Unsociability did not significantly predict anxiety at low levels of age (B_U-an_ = 0.047, p = 0.737), but had a significant negative predictive effect on anxiety at high levels of age (B_U-an_ = -0.641, p = 0.013).

## 4 Discussion

### 4.1 Key findings: redefining social withdrawal in mental health

This study reexamines social withdrawal through a subtype-specific lens in a large Chinese sample. Consistent with prior findings, shyness and avoidance—withdrawal patterns marked by social fear or avoidance—were associated with elevated internalizing symptoms and lower well-being. In contrast, unsociability was unrelated to psychological distress and, particularly among older participants, positively associated with well-being. These results contribute to evidence suggesting that social withdrawal patterns may not be uniformly maladaptive and align with emerging evidence distinguishing volitional solitude from anxiety-driven withdrawal. Notably, the finding that unsociability’s association with well-being is moderated by age reinforces calls to adopt a developmental timing perspective on solitude (3).

### 4.2 Theoretical implications for developmental psychiatry

These findings deepen developmental theory by foregrounding motivational differences in withdrawal behaviors. Anxiety-linked withdrawal (shyness/avoidance) remains maladaptive (3, 4), whereas unsociability may indicate self-determined solitude, particularly during identity consolidation stages. This resonates with the dual-pathway model (3) and lifespan development frameworks like Erikson’s psychosocial theory. Moreover, our results echo Yuan et al. (12), who found that depression mediates the relationship between shyness/avoidance and sleep problems, but not for unsociability, suggesting heterogeneous affective pathways across subtypes. These insights advocate for process-oriented psychiatric models that differentiate withdrawal motivations and their age-sensitive functions.

### 4.3 Cultural psychiatry perspectives: collectivistic contexts

Our findings provide empirical support for the cultural contingency of social withdrawal’s implications. Specifically, the negative association between unsociability and anxiety among older participants resonates with normative developmental expectations within Chinese culture. The Confucian ideal of “三十而立” (thirty as the age of establishment) emphasizes emotional maturity, self-discipline, and responsibility by early adulthood. In this context, a preference for solitude in one’s early thirties may be interpreted not as social failure, but as a sign of introspection, autonomy, or self-sufficiency, especially when it does not disrupt relational harmony. This cultural framework helps explain why unsociability was linked to lower anxiety only among older individuals in our sample, suggesting that age-related social expectations shape whether solitude is experienced as adaptive or problematic.

While we did not measure cultural values directly, this interpretation aligns with emerging cross-cultural evidence. For example, our results are consistent with Galanaki et al. (10), who found unsociability to be associated with lower existential anxiety in Greek emerging adults, and with Zhu et al. (11), who highlighted the role of resilience in moderating unsociability’s outcomes among Chinese migrant children. Together, these studies underscore that the meaning of social withdrawal is not fixed but is filtered through cultural and developmental lenses. Our study extends this literature by demonstrating that within a single cultural context, age itself can serve as a proxy for shifting cultural expectations, thereby altering the psychological impact of unsociability.

### 4.4 Clinical translation and mental health practice

These results challenge clinical tendencies to pathologize social withdrawal indiscriminately. Clinicians should distinguish between avoidable/shy withdrawal—linked to emotional distress—and unsociability, which may denote normative or even beneficial solitude. This aligns with research on “positive solitude” as a functional and adaptive state (10). Assessment tools and interventions should incorporate motivational context, age stage, and cultural meaning, rather than focusing solely on social engagement frequency. Such a nuanced approach promotes ecological validity and targets youth who truly need support, while respecting culturally and developmentally normative patterns.

The implications of our findings are particularly salient in today’s media environment. As emerging adults increasingly navigate social life through digital platforms, the line between social withdrawal and online social preference has become blurred. For instance, individuals who exhibit unsociability offline may still actively participate in online forums, maintain digital friendships, or express themselves in virtual communities. Prior research has shown that unsociable individuals may gravitate toward low-demand or asynchronous forms of online interaction, thereby fulfilling social needs without violating their preference for solitude (6). These insights suggest that interventions targeting social withdrawal should consider the medium through which sociality is expressed. Rather than assuming withdrawal from face-to-face interaction implies social dysfunction, practitioners should evaluate the role of new media as a compensatory or even preferred form of connection in this population.

### 4.5 Study limitations and future research priorities

This work has several constraints:

1. Cross-sectional design precludes causal inference. Longitudinal studies are required to track how withdrawal motives and outcomes shift across development.
2. We did not measure cultural values, role expectations, or internalized norms—these unmeasured variables may mediate moderation effects. Future studies integrating such constructs would enhance explanatory power.
3. Reliance on self-report data raises concerns of bias. Incorporating multi-informant methods or momentary assessments would improve validity. Furthermore, although we sought a diverse sample, our use of an online paid survey platform means our participants may not be fully representative of the broader Chinese emerging adult population, potentially limiting the generalizability of our findings.
4. We focused on psychological outcomes; future research should integrate physical health indicators, such as sleep and somatic symptoms (12).
5. In the New Media environment, individuals’ social behavior patterns are undergoing profound changes. While this study initially conceptualized New Media as a broader social context, it did not systematically examine how usage patterns and frequencies across specific platforms may differentially influence subtypes of social withdrawal and their associated mental health outcomes. Future research should systematically explore this complex interplay, thereby advancing a more nuanced understanding of the role of New Media use in shaping social withdrawal behaviors.

Taken together, these findings underscore the need for developmentally and culturally contextualized frameworks when conceptualizing social withdrawal. Moving beyond universal risk models to more nuanced appraisals can enrich theory and inform mental health policies attuned to diverse populations.

## Data Availability

The raw data supporting the conclusions of this article will be made available by the authors, without undue reservation.
